# Investigation of social, demographic and health variations in the usage of prescribed and over-the-counter medicines within a large cohort (South Yorkshire, UK)

**DOI:** 10.1136/bmjopen-2016-012038

**Published:** 2016-09-28

**Authors:** Mark A Green, Emma Little, Richard Cooper, Clare Relton, Mark Strong

**Affiliations:** 1 Department of Geography and Planning, University of Liverpool, Liverpool, UK; 2 School of Health and Related Research (ScHARR), University of Sheffield, Sheffield, UK

**Keywords:** SOCIAL MEDICINE, PUBLIC HEALTH, EPIDEMIOLOGY

## Abstract

**Objectives:**

Prescribed and over-the-counter (non-prescribed) medicine usage has increased in recent years; however, there has been less investigation of the socioeconomic predictors of use. This has been due to a lack of data, especially for over-the-counter medicines. Our study aims to understand how prescribed and over-the-counter medicine patterns vary by demographic, social and health characteristics within a large population cohort.

**Design:**

Cross-sectional data analysis.

**Setting:**

South Yorkshire, UK.

**Participants:**

27 806 individuals from wave 1 of the Yorkshire Health Study (2010–2012).

**Measures:**

Individuals self-reported each medicine they were taking and whether each was prescribed or not. The medicines were grouped into 14 categories (eg, cardiovascular system, infection, contraception). Negative binomial regression models were used to analyse the count of medicine usage. We included demographic (age, gender, ethnicity), social (education), health-related (body mass index, smoking, alcohol consumption, physical activity) factors and chronic health conditions (eg, stroke, anxiety and heart disease) in our analyses.

**Results:**

49% of men and 62% of women were taking medicine with the majority of this prescribed (88% and 83%, respectively). Health conditions were found to be positively associated with prescribed medicine usage, but mixed in their associated with over-the-counter medicines. Educational attainment was negatively associated with prescribed and positively associated with over-the-counter usage.

**Conclusions:**

Our study addresses a dearth of evidence to provide new insights into how behaviours in medicine usage vary by demographic, social and health-related factors. Differences in over-the-counter medicine usage by educational attainment may help our understanding of the determinants of health inequalities.

**Strengths and limitations of this study**

- We address a dearth of evidence on socioeconomic differences in terms of prescribed and over-the-counter (non-prescribed) medication usage (disaggregated by medicine type) using a large secondary data set.
- Our data are self-reported and may be subject to bias suggesting the need for objective data.
- Our analysis is only cross-sectional and extending this investigation longitudinally will be necessary for assessing the importance of our results.

## Introduction

There have been recognised increases in the production, affordability and consumption of medicines globally for many years,^1^ reflecting a market of several hundreds of billions of pounds. In England, for example, the total volume of medicine taken across England has grown with the total number of medicines prescribed by general practitioners (GPs) tripling in the 15 years up to 2010.^2^ This growth has occurred during a period of rising life expectancy, quality of life and better healthcare.

Over-the-counter (ie, non-prescribed and not funded by the state) medicines represent the other key supply route, and this also represents a significant market, with over 900 million packs being supplied in the UK in 2011.^3^ Indeed, the cost of medicines has been the subject of increasing attention, where it represents a key burden in healthcare systems like the National Health System (NHS) in the UK. Over-the-counter medicine use has been argued to represent a potential saving by reducing NHS spending by shifting the financial burden to individuals.^4^ However, the approach also follows a wider NHS strategy to enhance patient's empowerment through promoting self-medication contrary to other European nations.^5–7^

Previous research into predictors of medicine usage has focused on demographic factors, particularly age and gender. Prescribed medication usage has been shown to increase with age due to the association between ill health and age,^8–11^ although childhood and adolescence also represent important focal points of research.^12^ ^13^ In contrast, over-the-counter medicine usage decreases with age.^9^ ^11^ ^14^ Differences in terms of gender have also been explored extensively, with women consistently found to use greater prescribed and over-the-counter medicines.^9–11^ ^14–16^ Women are more health conscious than men, and have greater interactions with healthcare systems, which might explain these differences.^17^ There has also been some investigation of gender inequity in medication usage in low-income and medium-income countries.^17^ ^18^

There have been fewer studies that have explored the influence of social determinants on medicine usage, particularly for over-the-counter medicines. This is despite a more extensive literature on social inequalities in health, health-related behaviours and health service usage.^19^ Inconsistent findings have been reported for patterns by prescribed medicines usage, although this appears dependent on the policy context.^9–12^ ^15^ ^20^ ^21^ The association for over-the-counter medicine usage appears clearer, with greater usage among individuals of higher social standing.^9^ ^11^ ^14^ ^21^ However, there has been less investigation of the social determinants of over-the-counter medicine due to a lack of available data. Understanding differences in medicine usage is important in explaining the existence of social inequalities in health.

There has been less investigation of population-level associations within the UK, with most of the evidence base conducted in the USA. This has been due to a lack of available data, particularly for over-the-counter medicines which represent important areas due to their potential benefits (saving GP time and NHS costs) and problems (medicine interactions, side effects, misuse and abuse^22^). Data sources often contain small sample sizes, restricting the generalisability of findings. While the Health Survey of England is the largest national survey on health behaviours in England, it only contains information on prescribed medicines.^23^ There has also been a concentration of research exploring the uptake of cardiovascular medicine,^24^ ^25^ ignoring potential variations in other types of medicines.

Our study aims to understand how prescribed and over-the-counter medicine patterns vary by demographic, social and health characteristics within a large population cohort. We also disaggregate our analyses by medicine type.

## Methods

### Data

Data were taken from the first wave (2010–2012) of the Yorkshire Health Study (formerly the South Yorkshire Cohort), a longitudinal observational survey.^26^ The first wave contained information on 27 806 individuals aged 16 and over that consisted of the South Yorkshire region of England. Data were self-reported by individuals. Records with missing medicine data were dropped, leaving a total analytical sample of 18 272.

Individuals were asked to record any medicines they were currently taking, including whether it was prescribed or not. The data were then grouped into 14 categories (linked to the British National Formulary (BNF)), loosely based on the area and organ system of the body being targeted by the medicine (table 1). This grouping system was selected to minimise overlap between categories and follows previous research.^8^ Names of groups follow the BNF other than ‘Malignant disease’ medication which we refer to as ‘chemotherapy/immunosuppressant’ as it is a more useful descriptor. We separated oral contraceptives and diabetes medicine from other endocrine system agents. Oral contraceptives were considered separately to be able to explore gender variations in endocrine system medicine usage.^16^ Diabetes medicines were also separated due to their high prevalence and relative importance in public health decision-making.

**Table 1:** Summary statistics on medicine usage, including the percentage of each category taken, the mean number taken and the percentage of each prescribed (n=18 272)

	Individuals taking each medicine (%)		Mean number of medicines taken*		Percentage of medicines prescribed (%)	
Category	Male	Female	Male	Female	Male	Female
Cardiovascular system	24.3	21.3	2.7	2.4	97.7	97.0
Gastrointestinal system	12.1	14.6	1.2	1.3	94.9	92.4
CNS (central nervous system)	9.6	16.0	1.3	1.3	92.5	90.4
CNS pain	15.5	21.4	1.5	1.5	83.6	79.4
Respiratory system	8.1	8.4	1.8	1.9	99.1	99.3
Infection	2.9	2.7	1.6	1.1	94.7	96.7
Endocrine system	3.4	9.3	1.2	1.2	99.6	98.5
Contraception	0.0	7.5	0.0	1.0	NA	97.3
Chemotherapy/immunosuppressant	0.2	0.3	1.3	1.2	100	100
Musculoskeletal system	4.5	9.8	1.2	1.3	63.1	74.6
Eye	1.8	2.5	1.3	1.3	96.5	91.6
Allergy	4.2	4.6	1.2	1.1	78.3	79.4
Weight loss	0.4	0.5	1.0	1.0	71.4	83.6
Smoking cessation	0.1	0.1	1.1	1.0	100	91.9
Dietary supplements	9.3	17.3	1.5	1.5	27.7	25.5
Skin	3.1	4.1	1.5	1.4	92.2	85.1
Diabetes	4.1	2.6	1.5	1.4	99.3	99.8
Genito-urinary system	3.3	1.4	1.1	1.0	94.4	99.0
Gout	1.6	0.3	1.2	1.2	96.8	98.6
Other	2.6	3.5	1.1	1.1	72.5	47.1
Any category	49.3	62.2	3.7	3.6	88.1	82.5

Estimates weighted by age, gender, deprivation.

*Individuals not taking each medicine excluded.

Demographic factors included in our analysis were age, gender and ethnicity since variations in medicine use with respect to these factors have been previously demonstrated.^9^ ^10^ ^14^ ^27^ Age was measured as a continuous variable (in years) and gender was measured as a binary variable (male or female). Ethnicity was dichotomised into White or Caucasian. We did not disaggregate the Caucasian category further due to the lack of heterogeneity in the sample (ie, 5.9% of the sample were Caucasian).

Education was the only social measure included. Education has been used as a proxy for socioeconomic status in previous research of medicine usage,^10^ ^11^ ^20^ ^21^ since a higher level of education allows individuals to access better employment opportunities and therefore maximise their socioeconomic status.^28^ ^29^ Education also captures human capital which may influence health-related behaviours through greater cognitive ability to engage with health promotion resources. Education was defined using the following groups (European Qualifications Framework (EQF) level provided); ‘no formal education’ (EQF level 1), ‘secondary level of education’ (GCSE (General Certificate of Secondary Education) level or equivalent; EQF level 2–3), ‘postsecondary level’ (A-level or equivalent; EQF level 4) and ‘degree level or higher’ (EQF level 5+).

Heath-related behaviours were captured using body weight, smoking, alcohol and physical activity behaviours. Body mass index (BMI) was used as a measure of relative body weight since it has been shown to be positively associated with medicine usage.^30^ BMI is calculated by dividing an individual's weight (kg) by their height squared (m^2^). Smoking status, alcohol consumption and physical activity level were all included since they are important predictors of health.^31–37^ Smoking status refers to whether an individual currently smokes or not. Alcohol consumption was measured as the number of units of alcohol consumed per week. Physical activity was measured using two variables: level of walking and level of physical exercise (eg, sport, gym). Each measure was categorised as: ‘none’, ‘<1 hour’, ‘1–3 hours’ or ‘>3 hours’ per week.

We also examined the role of 12 chronic health conditions. Individuals reported whether they had any of the following long-standing conditions or disabilities; fatigue, pain, insomnia, anxiety, depression, diabetes, breathing problems (eg, chronic bronchitis), high blood pressure, heart disease, osteoarthritis, stroke or cancer. These were each individually included as explanatory variables.

Each GP practice was included as a separate variable in the analysis in order to account for differences in prescribing patterns between surgeries (results not reported due to the large number of surgeries).^20^

### Statistical analysis

The prevalence of each medicine category was reported and weighted using sample weights. Weighting was necessary because the Yorkshire Health Study contains some bias since it is over-representative of the elderly, females and individuals from affluent areas.^26^ Weighting allowed us to correct for known bias (sample weights were not used in the regression models). Analysis of medicine usage was conducted using total medicines split by prescription status. Medicine data were considered to have a Poisson distribution; however, variances were greater than mean values. To account for the overdispersion, negative binomial regression models were used to analyse medicine usage. Incident rate ratios (IRR) and their 95% CIs were reported. All explanatory variables were included in each multivariate model. GP practice could not be included as a random effect in the model since it resulted in the model becoming unstable and unable to converge. The analysis was also repeated for individual medicine categories to explore differences between them. Only the most prevalent medicine types (a sample size >10%; table 1) were selected to avoid small sample size issues. All analyses were undertaken using STATA/SE V.13.0.

## Results

Table 1 presents a summary of self-reported medicines taken split by category and gender. A greater proportion of women (62.2%) were found to be taking any category of medicine in comparison to men (49.3%). However, there was little difference in the mean number of any medicine category taken. Cardiovascular system medicines were the most common medicine taken, with gastrointestinal system, CNS (central nervous system), CNS pain and dietary supplements also commonly used. There was little difference in the mean number of medicines taken split by category or gender. The majority of medicines taken were prescribed; however, the proportion prescribed varied by category. Dietary supplements were the only category with greater over-the-counter medicines than prescribed medicines.

Table 2 presents the results from the regression models exploring the association of our variables to the number of prescribed and over-the-counter medicines taken. Age was positively associated with greater prescribed and over-the-counter medicine usage. Women were more likely to take prescribed and over-the-counter medicines. Although individuals from ethnic minority groups were less likely to use prescribed medicine than White individuals, there were no differences for over-the-counter medicine. BMI was positively associated with the number of prescribed medicines but unrelated to over-the-counter medicine.

**Table 2:** Results from negative binomial regression models analysing the associated factors of medicine usage, split by medicine type (n=18 272)

	Total prescribed	Total over-the-counter
Variable	IRR (95% CI)	IRR (95% CI)
Demographic		
Age	1.025*** (1.024 to 1.027)	1.027*** (1.024 to 1.030)
Male	0.894*** (0.863 to 0.925)	0.600*** (0.553 to 0.651)
Caucasian	0.756*** (0.670 to 0.853)	0.805 (0.628 to 1.033)
Social		
*Education*		
None	Reference	Reference
Secondary	0.943** (0.904 to 0.985)	1.665*** (1.495 to 1.856)
Postsecondary	1.013 (0.951 to 1.080)	1.817*** (1.573 to 2.099)
Degree or higher	0.950* (0.906 to 0.997)	1.967*** (1.754 to 2.206)
Health-related		
Body mass index	1.011*** (1.007 to 1.014)	1.001 (0.993 to 1.009)
Units of alcohol	0.995*** (0.993 to 0.996)	1.000 (0.996 to 1.004)
Smoker	1.065* (1.013 to 1.120)	0.786*** (0.695 to 0.888)
*Walking (per week)*		
None	Reference	Reference
<1 hour	1.001 (0.933 to 1.074)	1.165 (0.957 to 1.418)
1–3 hours	0.976 (0.914 to 1.042)	1.355** (1.131 to 1.624)
3+ hours	0.938 (0.879 to 1.001)	1.500*** (1.253 to 1.795)
*Physical exercise (per week)*		
None	Reference	Reference
<1 hour	0.947 (0.891 to 1.006)	1.156* (1.011 to 1.323)
1–3 hours	0.926** (0.884 to 0.971)	1.188** (1.074 to 1.313)
3+ hours	0.854*** (0.811 to 0.898)	1.231*** (1.106 to 1.370)
Chronic health		
Fatigue	1.208*** (1.154 to 1.265)	1.052 (0.933 to 1.185)
Pain	1.528*** (1.467 to 1.593)	1.563*** (1.412 to 1.730)
Insomnia	1.007 (0.947 to 1.070)	1.116 (0.957 to 1.302)
Anxiety	1.155*** (1.093 to 1.221)	1.064 (0.925 to 1.224)
Depression	1.449*** (1.365 to 1.537)	1.141 (0.978 to 1.331)
Diabetes	1.807*** (1.708 to 1.912)	0.747** (0.628 to 0.888)
Breathing problems	1.949*** (1.861 to 2.041)	0.972 (0.858 to 1.101)
High blood pressure	1.907*** (1.835 to 1.983)	0.863** (0.776 to 0.959)
Heart disease	1.824*** (1.723 to 1.931)	0.771** (0.647 to 0.920)
Osteoarthritis	1.143*** (1.086 to 1.203)	1.280*** (1.127 to 1.453)
Stroke	1.259*** (1.146 to 1.384)	0.600** (0.438 to 0.824)
Cancer	1.276*** (1.174 to 1.387)	0.850 (0.676 to 1.069)
Constant	0.220*** (0.188 to 0.257)	0.054*** (0.037 to 0.078)
/ln α	− 0.761	1.178
α	0.467	3.249
Pseudo r^2^	0.154	0.039

GP surgery was also adjusted for, including each surgery in the model as binary variables but not included in the table.

Significance levels: *<0.05, **<0.01, ***<0.001.

IRR, incident rate ratio.

The chronic illness and health conditions variables were consistently positively associated with greater prescribed medicines taken, with only insomnia having no significant relationship. Diabetes, breathing problems, high blood pressure and heart disease had stronger associations than anxiety, stroke and fatigue. Negative associations were found for the relationships between over-the-counter medicine use and diabetes, high blood pressure, stroke and heart disease. Pain and osteoarthritis were significantly and positively associated with prescribed and over-the-counter medicine use.

Consumption of alcohol was negatively associated with the number of prescribed medicines taken, but smoking was associated with increased number of prescribed medicines. For over-the-counter medicine, there was no significant association for alcohol, whereas the relationship for smoking was reversed. Walking was not associated with prescribed medicine, but positively associated with over-the-counter medicine. Physical exercise followed a similar pattern to walking for over-the-counter medicines, but the relationship reversed for prescribed medicine. Higher education levels were each negatively associated with total prescribed medicine (in comparison to the ‘no qualification’ category), although the strength of each association was weak. This contrasted with over-the-counter medicine, where education was positively associated with the use of over-the-counter medicines.

Tables 3 and 4 present the results of the negative binomial regression for prescribed and over-the-counter medicines, respectively. There were fewer significant associations; however, the results mostly followed the findings from table 2, particularly for age, gender and education. We observed some large effect sizes for some chronic health conditions to prescribed medicines associated with treating the condition (eg, cardiovascular system medicine and individuals reporting high blood pressure (IRR=4.205, 95% CIs 3.995 to 4.425)). These associations were not always immediately obvious, with depression strongly associated with CNS medicine (IRR=4.210, 95% CIs 3.770 to 4.700) and fatigue associated with dietary supplements (IRR=2.273, 95% CIs 1.877 to 2.752). Similar associations were not observed for chronic health conditions and over-the-counter medicines, although chronic pain was significantly positively associated with each medicine type apart from cardiovascular medicine. Some chronic health conditions were also negatively associated with medicine usage (eg, diabetes and gastrointestinal system medicine; IRR=0.327, 95% CIs 0.142 to 0.752).

**Table 3:** Results of negative binomial regressions analysing prescribed medicine usage (n=18 272)

	Cardiovascular system	Gastrointestinal system	CNS	CNS pain	Musculoskeletal system	Dietary Supplements
Variable	IRR (95% CI)	IRR (95% CI)	IRR (95% CI)	IRR (95% CI)	IRR (95% CI)	IRR (95% CI)
Demographic						
Age	1.054*** (1.052 to 1.056)	1.031*** (1.028 to 1.035)	1.013*** (1.009 to 1.016)	1.017*** (1.014 to 1.020)	1.056*** (1.049 to 1.062)	1.024*** (1.018 to 1.030)
Male	1.298*** (1.233 to 1.365)	0.897** (0.830 to 0.970)	0.694*** (0.632 to 0.763)	0.872*** (0.812 to 0.936)	0.440*** (0.381 to 0.509)	0.704*** (0.599 to 0.827)
Caucasian	0.723** (0.583 to 0.895)	0.523*** (0.369 to 0.742)	0.770 (0.570 to 1.041)	0.713* (0.546 to 0.931)	0.768 (0.445 to 1.325)	0.869 (0.507 to 1.489)
Social						
*Education*						
None	Reference	Reference	Reference	Reference		
Secondary	0.955 (0.899 to 1.015)	0.974 (0.886 to 1.070)	0.959 (0.856 to 1.075)	1.099* (1.009 to 1.196)	0.915 (0.775 to 1.080)	0.954 (0.786 to 1.159)
Postsecondary	0.936 (0.846 to 1.036)	0.987 (0.851 to 1.145)	0.943 (0.798 to 1.115)	1.173* (1.032 to 1.333)	0.927 (0.711 to 1.209)	1.084 (0.814 to 1.443)
Degree or higher	0.856*** (0.800 to 0.917)	0.966 (0.867 to 1.077)	0.893 (0.782 to 1.020)	0.985 (0.888 to 1.093)	1.008 (0.837 to 1.213)	0.847 (0.677 to 1.061)
Health-related						
Body mass index	1.032*** (1.027 to 1.037)	1.012** (1.005 to 1.019)	1.004 (0.996 to 1.012)	1.019*** (1.013 to 1.025)	0.962*** (0.949 to 0.975)	0.980** (0.965 to 0.994)
Units of alcohol	0.999 (0.997 to 1.002)	0.998 (0.994 to 1.002)	0.987*** (0.982 to 0.991)	0.991*** (0.988 to 0.995)	0.982*** (0.973 to 0.990)	0.985** (0.977 to 0.994)
Smoker	1.167*** (1.079 to 1.261)	0.934 (0.832 to 1.049)	1.200** (1.066 to 1.350)	1.303*** (1.187 to 1.430)	1.113 (0.907 to 1.365)	1.320* (1.067 to 1.633)
*Walking (per week)*						
None	Reference	Reference	Reference	Reference	Reference	Reference
<1 hour	1.153** (1.045 to 1.273)	0.972 (0.849 to 1.114)	0.734*** (0.629 to 0.856)	0.897 (0.799 to 1.008)	1.049 (0.814 to 1.352)	0.821 (0.616 to 1.093)
1–3 hours	1.176** (1.072 to 1.289)	0.931 (0.820 to 1.058)	0.639*** (0.552 to 0.738)	0.777*** (0.696 to 0.868)	0.871 (0.686 to 1.106)	0.761* (0.583 to 0.994)
3+ hours	1.162** (1.059 to 1.274)	0.867* (0.762 to 0.987)	0.556*** (0.480 to 0.644)	0.756*** (0.676 to 0.845)	0.836 (0.658 to 1.062)	0.671** (0.513 to 0.877)
*Physical exercise (per week)*						
None	Reference	Reference	Reference	Reference	Reference	Reference
<1 hour	0.955 (0.869 to 1.050)	0.884 (0.765 to 1.022)	0.834* (0.707 to 0.983)	0.972 (0.857 to 1.102)	0.935 (0.730 to 1.197)	1.000 (0.761 to 1.315)
1–3 hours	0.919 (0.854 to 0.989)	0.880* (0.787 to 0.985)	0.764*** (0.669 to 0.872)	0.859** (0.774 to 0.953)	1.032 (0.860 to 1.237)	0.892 (0.715 to 1.113)
3+ hours	0.927 (0.858 to 1.002)	0.787*** (0.692 to 0.896)	0.633*** (0.538 to 0.744)	0.720*** (0.634 to 0.817)	0.873 (0.703 to 1.083)	0.881 (0.686 to 1.130)
Chronic health						
Fatigue	1.017 (0.953 to 1.085)	1.503*** (1.369 to 1.649)	1.213** (1.084 to 1.357)	1.099* (1.012 to 1.193)	1.646*** (1.393 to 1.943)	2.273*** (1.877 to 2.752)
Pain	1.047 (0.986 to 1.112)	1.833*** (1.678 to 2.003)	1.534*** (1.378 to 1.708)	5.088*** (4.695 to 5.514)	1.952*** (1.671 to 2.280)	1.232* (1.021 to 1.487)
Insomnia	0.954 (0.873 to 1.042)	1.017 (0.907 to 1.141)	0.926 (0.812 to 1.056)	1.060 (0.961 to 1.170)	1.069 (0.868 to 1.317)	0.949 (0.739 to 1.220)
Anxiety	1.109* (1.021 to 1.204)	1.178** (1.056 to 1.314)	2.138*** (1.912 to 2.390)	0.936 (0.850 to 1.032)	0.867 (0.704 to 1.066)	1.169 (0.925 to 1.478)
Depression	1.048 (0.957 to 1.147)	1.215** (1.081 to 1.366)	4.210*** (3.770 to 4.700)	1.312*** (1.186 to 1.451)	1.170 (0.933 to 1.467)	1.237 (0.964 to 1.587)
Diabetes	1.675*** (1.563 to 1.795)	0.895 (0.791 to 1.013)	0.965 (0.823 to 1.132)	0.878* (0.783 to 0.985)	0.955 (0.754 to 1.208)	1.613*** (1.258 to 2.068)
Breathing problems	0.983 (0.917 to 1.054)	1.237*** (1.123 to 1.362)	0.929 (0.821 to 1.052)	1.029 (0.941 to 1.124)	1.314** (1.103 to 1.566)	1.186 (0.962 to 1.461)
High blood pressure	4.205*** (3.995 to 4.425)	1.076 (0.989 to 1.172)	0.970 (0.868 to 1.085)	1.013 (0.937 to 1.096)	0.876 (0.750 to 1.022)	1.154 (0.962 to 1.384)
Heart disease	3.330*** (3.115 to 3.560)	1.377*** (1.236 to 1.535)	1.099 (0.940 to 1.284)	1.087 (0.977 to 1.210)	1.105 (0.893 to 1.367	1.232 (0.957 to 1.588)
Osteoarthritis	0.973 (0.909 to 1.042)	1.216*** (1.105 to 1.339)	0.978 (0.858 to 1.114)	1.720*** (1.587 to 1.866)	1.653*** (1.402 to 1.949)	1.099 (0.883 to 1.368)
Stroke	1.631*** (1.463 to 1.818)	1.097 (0.921 to 1.308)	1.238 (0.994 to 1.543)	1.002 (0.846 to 1.186)	1.012 (0.722 to 1.418)	0.912 (0.603 to 1.380)
Cancer	0.964 (0.862 to 1.078)	1.386*** (1.194 to 1.609)	0.959 (0.766 to 1.120)	1.127 (0.969 to 1.311)	1.290 (0.977 to 1.702)	1.325 (0.943 to 1.860)
Constant	0.005*** (0.003 to 0.006)	0.018*** (0.013 to 0.026)	0.085*** (0.058 to 0.125)	0.033*** (0.024 to 0.044)	0.010*** (0.005 to 0.018)	0.028*** (0.014 to 0.055)
ln α	−0.906	−1.327	−0.628	−1.175	0.909	1.344
α	0.404	0.265	0.534	0.309	2.481	3.836
Pseudo r^2^	0.284	0.142	0.176	0.208	0.144	0.082

GP surgery was also adjusted for, including each surgery in the model as binary variables but not included in the table.

Significance levels: *<0.05, **<0.01, ***<0.001.

CNS, central nervous system; IRR, incident rate ratio.

**Table 4:** Results of negative binomial regressions analysing over-the-counter medicine usage (n=18 272)

	Cardiovascular system	Gastrointestinal system	CNS	CNS pain	Musculoskeletal system	Dietary Supplements
Variable	IRR (95% CI)	IRR (95% CI)	IRR (95% CI)	IRR (95% CI)	IRR (95% CI)	IRR (95% CI)
Demographic						
Age	1.052*** (1.042 to 1.063)	1.033*** (1.023 to 1.044)	0.995 (0.985 to 1.004)	1.009** (1.003 to 1.015)	1.057*** (1.050 to 1.064)	1.031*** (1.027 to 1.034)
Male	0.963 (0.765 to 1.212)	0.505*** (0.377 to 0.675)	0.493*** (0.366 to 0.664)	0.597*** (0.509 to 0.701)	0.624*** (0.530 to 0.736)	0.573*** (0.519 to 0.633)
Caucasian	0.769 (0.304 to 1.947)	1.305 (0.591 to 2.877)	1.147 (0.569 to 2.314)	0.720 (0.433 to 1.196)	0.953 (0.501 to 1.814)	0.746 (0.542 to 1.027)
Social						
*Education*						
None	Reference	Reference	Reference	Reference	Reference	Reference
Secondary	1.465** (1.101 to 1.949)	3.162*** (2.155 to 4.639)	1.665* (1.126 to 2.463)	1.883*** (1.523 to 2.328)	1.700*** (1.380 to 2.094)	1.659*** (1.454 to 1.892)
Postsecondary	1.342 (0.859 to 2.095)	3.457*** (2.113 to 5.656)	2.346*** (1.476 to 3.730)	2.032*** (1.542 to 2.676)	1.486* (1.082 to 2.041)	1.806*** (1.514 to 2.155)
Degree or higher	1.539** (1.130 to 2.096)	2.862*** (1.878 to 4.361)	1.916** (1.253 to 2.928)	1.943*** (1.541 to 2.450)	2.051*** (1.651 to 2.549)	2.045*** (1.781 to 2.348)
Health-related						
Body mass index	1.036** (1.013 to 1.059)	0.989 (0.963 to 1.015)	1.034** (1.011 to 1.058)	1.021** (1.006 to 1.035)	1.004 (0.987 to 1.021)	0.986** (0.976 to 0.996)
Units of alcohol	1.003 (0.992 to 1.013)	1.004 (0.991 to 1.018)	1.000 (0.987 to 1.014)	1.000 (0.992 to 1.008)	1.007 (0.999 to 1.014)	0.998 (0.993 to 1.003)
Smoker	1.412* (1.016 to 1.961)	0.603* (0.379 to 0.962)	1.375 (0.987 to 1.915)	0.897 (0.717 to 1.122)	0.669* (0.490 to 0.913)	0.690*** (0.590 to 0.808)
*Walking (per week)*						
None	Reference	Reference	Reference	Reference	Reference	Reference
<1 hour	2.193* (1.141 to 4.214)	0.721 (0.379 to 1.372)	0.546 (0.267 to 1.115)	1.268 (0.885 to 1.816)	0.997 (0.647 to 1.536)	1.174 (0.916 to 1.505)
1–3 hours	2.291** (1.228 to 4.275)	1.250 (0.718 to 2.177)	1.305 (0.728 to 2.337)	1.375 (0.985 to 1.920)	1.218 (0.823 to 1.801)	1.403** (1.116 to 1.764)
3+ hours	3.106*** (1.676 to 5.758)	1.004 (0.574 to 1.756)	1.631 (0.918 to 2.896)	1.422* (1.021 to 1.982)	1.622* (1.104 to 2.384)	1.519*** (1.211 to 1.907)
*Physical exercise (per week)*						
None	Reference	Reference	Reference	Reference	Reference	Reference
<1 hour	1.308 (0.899 to 1.903)	1.045 (0.658 to 1.660)	0.962 (0.620 to 1.490)	0.963 (0.742 to 1.251)	1.259 (0.950 to 1.669)	1.158 (0.983 to 1.363)
1–3 hours	0.906 (0.660 to 1.244)	1.231 (0.894 to 1.694)	0.910 (0.652 to 1.271)	0.971 (0.800 to 1.180)	1.511*** (1.248 to 1.829)	1.227** (1.089 to 1.383)
3+ hours	1.029 (0.746 to 1.421)	1.190 (0.831 to 1.702)	0.760 (0.509 to 1.136)	0.939 (0.757 to 1.163)	1.645*** (1.351 to 2.003)	1.334*** (1.175 to 1.514)
Chronic health						
Fatigue	1.027 (0.741 to 1.425)	1.033 (0.720 to 1.483)	0.860 (0.592 to 1.249)	1.076 (0.872 to 1.327)	0.806 (0.627 to 1.035)	1.086 (0.940 to 1.254)
Pain	1.192 (0.896 to 1.585)	1.938*** (1.424 to 2.639)	2.765*** (2.027 to 3.772)	3.457*** (2.908 to 4.111)	1.449*** (1.192 to 1.763)	1.140* (1.005 to 1.293)
Insomnia	1.084 (0.715 to 1.645)	0.989 (0.642 to 1.522)	1.554* (1.031 to 2.342)	1.084 (0.834 to 1.408)	1.135 (0.843 to 1.528)	1.091 (0.907 to 1.311)
Anxiety	1.114 (0.756 to 1.640)	1.505* (1.031 to 2.197)	1.228 (0.830 to 1.816)	1.009 (0.792 to 1.286)	0.701* (0.509 to 0.963)	1.079 (0.911 to 1.277)
Depression	0.940 (0.601 to 1.471)	1.364 (0.888 to 2.094)	1.360 (0.902 to 2.052)	1.131 (0.871 to 1.469)	0.880 (0.612 to 1.266)	1.201 (0.996 to 1.447)
Diabetes	0.840 (0.559 to 1.263)	0.327** (0.142 to 0.752)	0.456* (0.210 to 0.994)	0.857 (0.616 to 1.194)	0.597* (0.403 to 0.883)	0.770* (0.623 to 0.952)
Breathing problems	0.615* (0.412 to 0.917)	1.121 (0.764 to 1.646)	0.772 (0.496 to 1.200)	0.991 (0.786 to 1.249)	0.925 (0.714 to 1.197)	1.042 (0.897 to 1.210)
High blood pressure	1.189 (0.922 to 1.533)	0.952 (0.684 to 1.326)	0.808 (0.548 to 1.192)	0.653*** (0.527 to 0.810)	0.682*** (0.557 to 0.834)	0.976 (0.861 to 1.106)
Heart disease	1.103 (0.751 to 1.618)	0.855 (0.491 to 1.491)	0.850 (0.423 to 1.709)	0.489*** (0.329 to 0.725)	0.599** (0.416 to 0.863)	0.861 (0.698 to 1.061)
Osteoarthritis	1.015 (0.729 to 1.412)	1.646** (1.165 to 2.324)	1.359 (0.909 to 2.031)	1.359** (1.088 to 1.700)	1.720*** (1.403 to 2.107)	1.268** (1.089 to 1.477)
Stroke	1.225 (0.664 to 2.262)	0.545 (0.170 to 1.749)	0.490 (0.119 to 2.025)	0.842 (0.471 to 1.504)	0.337* (0.138 to 0.822)	0.560** (0.374 to 0.840)
Cancer	0.624 (0.322 to 1.209)	0.421 (0.155 to 1.148)	1.514 (0.766 to 2.990)	0.903 (0.584 to 1.396)	1.134 (0.777 to 1.655)	0.840 (0.637 to 1.107)
Constant	0.000*** (0.000 to 0.000)	0.001*** (0.000 to 0.004)	0.004*** (0.001 to 0.012)	0.011*** (0.006 to 0.023)	0.001*** (0.000 to 0.002)	0.033*** (0.021 to 0.052)
ln α	0.954	−0.118	−35.436	1.382	−1.068	1.164
α	2.595	0.889	0.000	3.981	0.344	3.202
Pseudo r^2^	0.072	0.087	0.0829	0.062	0.110	0.047

GP surgery was also adjusted for, including each surgery in the model as binary variables but not included in the table.

Significance levels: *<0.05, **<0.01, ***<0.001.

CNS, central nervous system; IRR, incident rate ratio.

## Discussion

This study has demonstrated variations by demographic, social and health factors in prescribed and over-the-counter medicine usage within a large cohort. Prescribed medicine usage was associated with the presence of chronic health conditions and poor health-related behaviours. Taking over-the-counter medicine was associated with higher levels of education, and positive health behaviours. Gender was also important for the purchasing of both medicine type, although the effect size was larger for over-the-counter medicines. The patterns were fairly consistent when analysing by medicine type.

The main strength of the study is it addresses the dearth of evidence of social and demographic patterns in medicine usage at the population level, particularly for over-the-counter medicines. There are several limitations to our study. The analysis is cross-sectional and therefore cannot demonstrate causality. Future waves of the Yorkshire Health Study will allow the findings to be tested longitudinally, helping to strengthen conclusions and recommendations.^26^ Data collected in the Yorkshire Health Study were self-reported and this may lead to bias in estimates. For example, recall bias may lead to under-reporting of medications, particularly for over-the-counter medications which are taken sporadically or not always thought of as medicines (eg, vitamins). Medicine dosage was not measured in the data limiting our comparison of medicines. The categories used to group together medicines may also hide variations in patterns, particularly where medications may be used for different purposes, despite operating at the same organ system/area of the body. Finally, we only use one measure of socioeconomic status (education) and extending our analyses to additional measures such as income or occupation will help to improve our understanding socioeconomic behaviours in medicine usage.

While individuals of high education took fewer medications compared with individuals with no qualifications (table 2), the effect size was only small and medicine usage was influenced more strongly by health status and age. In contrast, a distinct social gradient in over-the-counter medicine usage was observed. These findings support similar results found in other countries.^11^ ^21^ Individuals of high education are associated with better employment prospects and higher incomes, and hence they will be in a better position to absorb the financial burden associated with purchasing additional medicines, particularly if it allows them to avoid long waiting times to see their GP.^9^ ^11^ ^21^ Education also incorporates an individual's ability to cognitively understand the potential benefits of over-the-counter medicines, as well as effectively communicate health information to clinicians.^38^ Alternative explanations for the role of education may include: differences in compliance to treatment, inequalities in access to pharmacies and variations in self-treatment behaviours.^10^ ^11^ Evaluating the contribution of these potential pathways is important for future research to be able to address social inequalities in health behaviours.

The relationships for physical exercise, walking and smoking may also be explained similarly to that of the cognitive role of education. Individuals who exercise regularly or do not smoke have been shown to have greater health consciousness.^39^ ^40^ Health consciousness may be captured through these variables in our analysis and it may be that these types of individuals also try to maximise their health using over-the-counter medicines. Given the association between high education and positive health-related behaviours throughout the literature,^19^ the role played by cognition appears important. This is contrary to the relationships with prescribed medicine where physical exercise is protective to health^35–37^ and smoking damaging,^31^ ^33^ ^34^ independently influencing the need for prescribed medicine. However, the results for alcohol consumption only followed this pattern for prescribed medicine.

Individuals who were Caucasian were found to be less likely to take prescribed medicines (but not over-the-counter medicines). Our results support evidence from the USA which has found similar associations of lower medicine usage among Caucasian individuals.^14^ ^41^ However, there is little understanding of why this association exists and therefore, further research should explore possible explanations, including social factors, access to healthcare or cultural factors. Addressing inequalities in healthcare usage by ethnicity will be important, given that most medicines are only available through prescriptions in the UK.

In summary, we find differences in prescribed and over-the-counter medicine usage by demographic, social and health characteristics. Education was an important factor in explaining variations in over-the-counter usage. With the NHS moving towards greater self-medication (to empower patients and reduce costs), such an approach may have important implications for social inequalities in health and health-related behaviours.
